# Improving efficiency in neuroimaging research through application of Lean principles

**DOI:** 10.1371/journal.pone.0205232

**Published:** 2018-11-28

**Authors:** Alexandra Roy, Julia Colpitts, Kara Becker, Judson Brewer, Remko van Lutterveld

**Affiliations:** 1 Center for Mindfulness, University of Massachusetts Medical School, Worcester, Massachusetts, United States of America; 2 Mindfulness Center, Brown University, Providence, Rhode Island, United States of America; 3 DynaMed Plus, EBSCO Health, Ipswich, Massachusetts, United States of America; Azienda Ospedaliera Universitaria Federico II, ITALY

## Abstract

**Introduction:**

“Lean” is a set of management principles which focus on increasing value and efficiency by reducing or avoiding waste (e.g., overproduction, defects, inventory, transportation, waiting, motion, over processing). It has been applied to manufacturing, education, and health care, leading to optimized process flow, increased efficiency and increased team empowerment. However, to date, it has not been applied to neuroimaging research.

**Methods:**

Lean principles, such as Value stream mapping (e.g. a tool with which steps in the workflow can be identified on which to focus improvement efforts), 5S (e.g. an organizational method to boost workplace efficiency and efficacy) and Root-cause analysis (e.g. a problem-solving approach to identify key points of failure in a system) were applied to an ongoing, large neuroimaging study that included seven research visits per participant. All team members participated in a half-day exercise in which the entire project flow was visualized and areas of inefficiency were identified. Changes focused on removing obstacles, standardization, optimal arrangement of equipment and root-cause-analysis. A process for continuous improvement was also implemented. Total time of an experiment was recorded before implementation of Lean for two participants and after implementation of Lean for two participants. Satisfaction of team members was assessed anonymously on a 5-item Likert scale, ranging from much worsened to much improved.

**Results:**

All team members (N = 6) considered the overall experience of conducting an experiment much improved after implementation of Lean. Five out of six team members indicated a much-improved reduction in time, with the final team member considering this somewhat improved. Average experiment time was reduced by 13% after implementation of Lean (from 142 and 147 minutes to 124 and 128 minutes).

**Discussion:**

Lean principles can be successfully applied to neuroimaging research. Training in Lean principles for junior research scientists is recommended.

## Introduction

Academic researchers constantly face pressures to produce external-facing results, including funded grants and publication of new research. With this constant drive and sense of urgency, they rarely turn a critical eye inward toward the internal operations and processes of their lab or a specific research protocol. This is not surprising due to time constraints and because scientists do not typically undertake training in operations or project management. Investigators who continuously evaluate and improve their operational processes will find a competitive advantage in terms of optimizing their use of time, space, staff, and funding. One method of continuous improvement is through the implementation of “Lean” methodologies.

Lean focuses on increasing value and efficiency by reducing or avoiding waste (e.g., overproduction, defects, inventory, transportation, waiting, motion, over processing). A single definition of Lean is elusive because of its broad applicability–it has been defined as a philosophy, a culture, a framework, as well as a set of management principles [1]. There are related schools of thought, such as Six Sigma, which focuses on addressing problems and reducing variation. In this paper, we will focus on Lean and the potential benefits of improving process flow and reducing waste in an academic research setting.

The term Lean was coined by the research team at Massachusetts Institute of Technology’s International Motor Vehicle Program to describe Toyota’s business model for automobile production [2]. The Toyota Production System was developed by Sakichi and Kiichiro Toyoda, along with engineer Taiichi Ohno, as post-war material supply limitations forced them to seek alternatives to Ford’s high-inventory assembly line model [3]. The result was a “just-in-time” model that includes the key principles of continuous improvement and respect for people. This model was depicted as a house conveying a foundation of operational stability with two pillars representing “just-in-time” manufacturing and built-in quality, which leads to the goals of quality, cost, safety, delivery, and morale as the roof. The “house of Toyota” was updated in The Toyota Way 2001 to reflect their values and business methods. The revised image depicts lean thinking as the foundation with two pillars representing continuous improvement and respect for people [4]. In the 1980s, Japanese auto producers required approximately 50% of the effort to assemble a car compared with European and American carmakers and that the quality of the former was 47% and 50% higher than European- and American-owned manufacturing plants, respectively [5]. These dramatic results highlight the benefits of this approach in manufacturing and the potential to achieve similar results in other industries.

Though Lean has its origin in manufacturing, more recently it has been adapted and applied to many industries, including software development, healthcare, laboratory science, and clinical and translational research. In healthcare, Lean principles have been particularly successful in addressing length of hospital stay (LOS). More specifically, several recent studies demonstrated that the application of Lean strategies helped reduce LOS post-surgery in patients in need of knee or hip replacements and reduced the number of patients who experience hospital associated infections [6–9]. In a case study evaluating Lean manufacturing principles applied to inpatient pharmacy drug dispensing, investigators found time savings in excess of 40% (measured as reduction in cycle time) [10]. Another study evaluated the use of Lean and Six Sigma in combination to reduce turnaround times for Emergency Department specimens. Investigators found multiple improved outcomes, including turnaround times and a reduction in unused specimens [11]. In a review of Lean methodology applied to Emergency Departments in the United States, Canada, and Australia, multiple positive impacts on team empowerment were found. This included “better awareness of their work and the problems therein”, “[becoming] empowered to suggest future changes”, and “new participative, continuous improvement culture” [12]. Similarly, a study evaluating the impact of Lean principles on employee empowerment in higher education found self-reported increases in efficiency, knowledge share, collaboration, and the ability to make decisions and implement change on processes or programs owned [13]. Its potential has also been recognized by the National Institutes of Health for the clinical and translational research enterprise [14]. The evolution and adaptation of Lean in different industries has resulted in making the case for terms like the “house of Lean”, “Lean Thinking”, a “Lean lifestyle”, and a “Lean journey” in order to more accurately reflect the cultural and organizational aspects of implementing Lean [15,16]. In neuroimaging research, however, this approach has received little to no attention. With the current drive to include more participants to boost validity in neuroimaging research, reducing inefficiencies is especially key [17].

This study aims to demonstrate how Lean principles can be applied to a neuroimaging study to reduce waste and empower the research team. Based on the results in other fields of work, we hypothesize that experiments will be conducted faster, and team satisfaction will increase after the implementation of Lean principles.

## Materials and methods

### Lean tools

There are a variety of Lean tools that can be leveraged to support process improvement initiatives and the choice of tool depends on the specific situation and goals. Commonly used Lean tools include 5S, A3, Kanban, Root-cause analysis, Failure Mode and Effects Analysis (FMEA), and Value stream mapping [18–21].

- **5S**: An organizational method for workplace efficiency and efficacy referring to five Japanese words that begin with “S” when transliterated into Roman script as well as when translated into English:
  - Sort: Eliminate obstacles and remove unnecessary items, e.g., ensuring that no unessential tasks are performed.
  - Set in order: Arrange necessary items and work station such that all equipment is in close proximity, e.g., making sure that all equipment in a lab is within arm’s reach in the lab.
  - Shine: Keep area clean and safe, e.g., keeping the lab clutter-free.
  - Standardize: Standardize orderliness, e.g., using a checklist to standardize the work across team members.
  - Sustain: Encourage self-discipline to maintain proper order, e.g., providing regular trainings to team members.
- **A3**: A problem-solving approach involving the use of an A3-sized paper template capturing a concise summary of a problem which is used as a communication tool to give structure to the improvement process. A3s can be utilized in four areas: problem solving, proposal, status report and strategic planning. An example of a status report A3 is a report showing the progress of a long-term research project on a quarterly basis showing planned recruitment vs actual status.
- **Kanban**: A Kanban system consists of visualizing the work, limiting the number of work items in progress, and focusing on the flow of work and continuous improvement. A simple example of this is to subdivide tasks of a project in categories “to do”, “in progress”, and “done”.
- **Root-cause analysis**: A problem-solving approach to identify key points of failure in a system. For example, understanding the root cause and solving the problem, instead of implementing work-around solutions, as these easily become ossified and introduce inefficiency. There are multiple root-cause analysis techniques, including:
  - Fishbone diagram: identifies many possible causes and sorts them into useful themes.
  - Pareto diagram: displays quantitative data in a bar graph to identify most significant factors.
- **Failure Mode and Effects Analysis**: A technique used to try to prevent errors by identifying all the ways a process could fail and proactively implementing changes to a system, design, process, and/or service. For example, creating a diagram that lists all the ways that data collection could fail and then making changes to prevent failure from happening.
- **Value stream mapping**: This entails visualizing the entire workflow, reaching a shared understanding of the current state, and, importantly, identifying steps in the workflow on which to focus improvement efforts to reach a desired future state. For example, mapping the entire workflow in an experiment from recruitment until the end of enrollment of a participant and identifying parts of the experiment which can be improved.

Importantly, these same tools can be used to foster continuous improvement after an initial implementation. Continuous improvement, representing one of the pillars of the “house of Lean,” can occur through additional incremental improvements to the same process or identification of a different process to focus on for improvement.

### Neuroimaging study

With the help of two external consultants who had training in Lean methodology (JC and KB), Lean principles were applied at the Therapeutic Neuroscience Lab located at the University of Massachusetts Medical School (UMMS) Center for Mindfulness on a large randomized controlled neurofeedback trial that was in its second year of data acquisition. The aim of this trial was to investigate whether real-time neurofeedback from the posterior cingulate cortex (PCC), which is a brain region that is involved in meditation quality, would change brain activity and be correlated with improvements in attention and mental health [22]. In this study, at baseline, 63 participants undergo a functional magnetic resonance imaging (fMRI) scan and cognitive testing and fill out several questionnaires. This is followed by five weekly electroencephalography (EEG) neurofeedback training sessions. After completion of the neurofeedback sessions participants repeat the baseline testing, then complete questionnaires at a 3-month follow-up. Each participant spends more than 16 hours interacting with a variety of members of the research team including the project leader, project director, fMRI technicians, EEG technicians, and post-doctoral fellows. EEG was used to provide the EEG neurofeedback sessions and fMRI was used to assess whether brain activity in the PCC was altered. EEG and fMRI are both techniques to measure directly (EEG) or indirectly (fMRI) brain activity, with the advantage of EEG that it measures brain activity on the millisecond time-scale, making it suitable to provide real-time neurofeedback, and the advantage of fMRI its spatial accuracy. Extended information on the procedure of the entire study is provided in supporting document, S1 File. The key question was: can Lean methodologies improve the efficiency, quality and overall satisfaction of this process?

### Value stream mapping

A value stream mapping exercise was selected as the initial tool to begin the Lean implementation because several team members were engaged in only specific aspects of the experiment. This enabled every team member to have the same overarching understanding of project flow. The entire team (10 people) participated in a half-day meeting in which the entire workflow was mapped from initial contact with the research participants to their completion of the study. Based on this workflow diagram, the team identified specific parts of the process where there were improvement opportunities and people were identified to investigate and implement changes.

**Fig 1:**
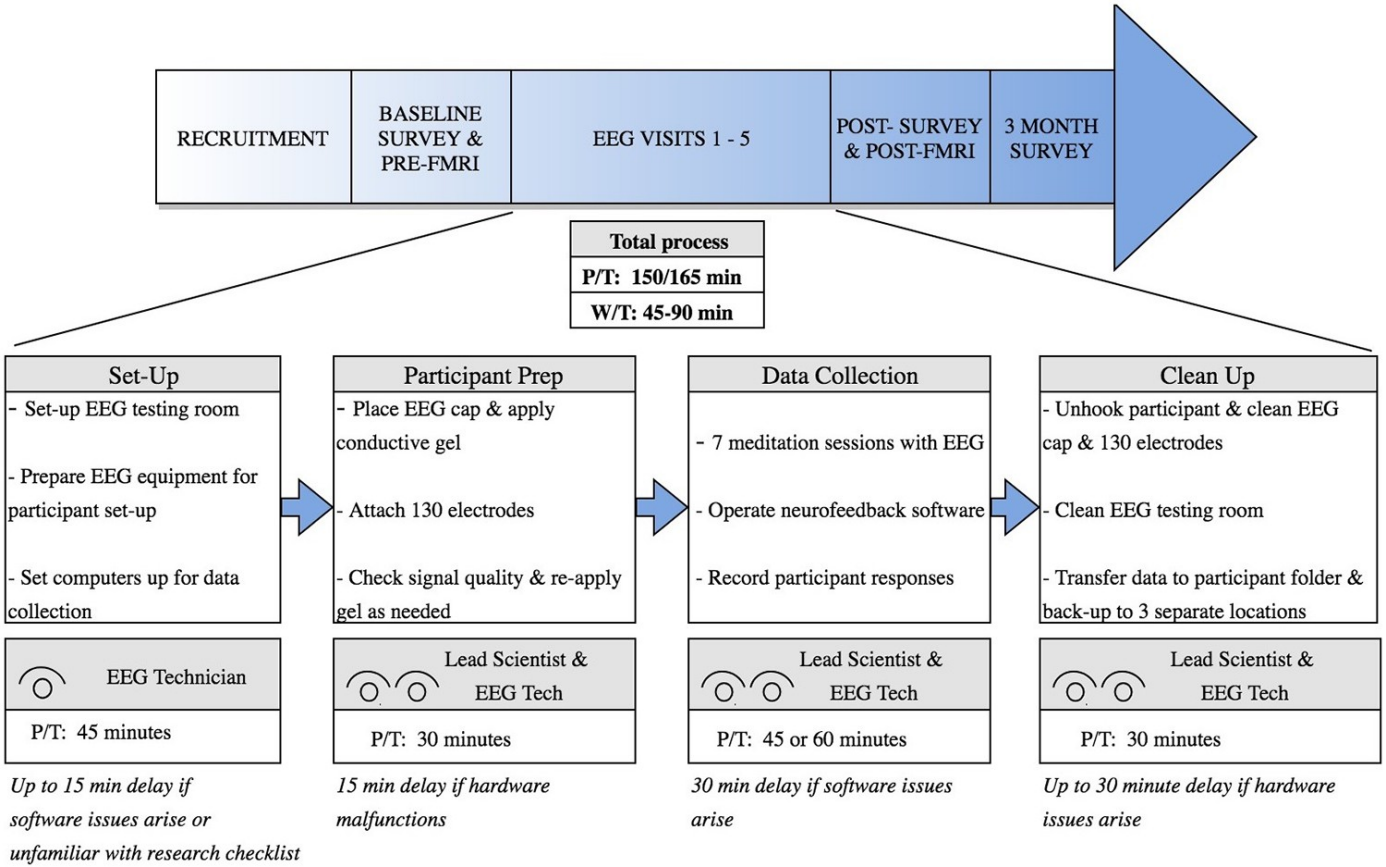
Value stream map depicting workflow of neurofeedback session (P/T: Process time, W/T: Wait time). fMRI: Functional magnetic resonance imaging. EEG: Electroencephalography.

During the value stream mapping exercise, the team decided to initially focus on the five EEG neurofeedback sessions because these were the most time-consuming component of the study for both the study participants and the research team. The neurofeedback sessions are conducted by a team of two people, with an EEG technician setting up the lab, operating the computers during the neurofeedback runs, and performing data migration and clean-up, and a research scientist interacting with the participant between runs. Fig 1 shows the resulting value stream map.

Three areas of improvement were identified that were predicted to have the greatest impact on the flow of an individual neurofeedback session:

1. The research checklist
2. Hardware and software
3. Organization of the lab itself

### The research checklist

One major change was updating and reformatting the 23-page research checklist. The checklist was initially developed more than one and a half years prior and had not been comprehensively updated to incorporate tips, shortcuts, and workarounds utilized by the research team. To make these updates, the checklist was reviewed by all team members and non-essential tasks were removed (5S: Sort). All checklist items were also reviewed and clustered in such a way that minimized the time spent physically moving from one task to the next on the list (5S: Set in order). In addition, the design of the list was updated to a two-column design; this was utilized to present key information and process steps in the left column and troubleshooting and additional tips with graphical explanation in the right column (5S: Standardize).

### Hardware and software

Additional EEG caps were purchased which eliminated the need to blow dry the EEG cap after cleaning between sessions (5S: Sort). In addition, an analysis was performed to understand why the neurofeedback software was occasionally unstable and crashed (Root-cause analysis). Based on this analysis, the architecture of the neurofeedback delivery was updated so it did not require the support of an intermediate software, which increased stability of the neurofeedback software and saved time. This meant that several work-arounds could be eliminated (5S: Sort).

### Lab organization

All items used in the study were relocated to areas that minimized the amount of walking the research team had to do during a neurofeedback session (5S: Set in order). For example, the storage location of the completed checklists and backup devices was moved to the EEG control room. In addition, a wireless mouse was acquired which made it possible to check EEG data quality from the experimental room during participant preparation, removing the necessity to walk to the control room. Furthermore, parts of the checklist that included scripted instructions were removed from the checklist, printed and laminated, which eliminated unnecessary printing and reduced the amount of paperwork (5S: Sort).

### Continuous improvement

The ultimate goal of Lean is to set up the team for continuous improvement. To achieve this goal, a debriefing protocol was implemented where team members reviewed each session at its conclusion to determine what was successful and where improvements could continue to be made. At the weekly lab meeting, these were reviewed and a decision on implementation was made.

### Effects of Lean implementation

To assess the effects of the Lean implementation, two measures were evaluated: total time of a neurofeedback session and team satisfaction. The total time of a neurofeedback session was recorded for two participants prior to Lean implementation and for two participants after Lean implementation. In addition, the satisfaction of the EEG neurofeedback team was assessed using an anonymous internet-based questionnaire. The first two questions assessed the overall experience using a 5-item Likert scale and the third question allowed for open-ended feedback.

Question 1: “On a scale of much worsened, somewhat worsened, the same, somewhat improved, much improved, how would you rate the changes in achieving the following goals by implementing Lean methodology: A) Reducing time spent on set-up, running, and clean-up of a neurofeedback session, B) Streamlining data capture and storage, C) Setting the team up for continuous improvement initiatives"

Question 2: “On a scale of much worsened, somewhat worsened, the same, somewhat improved, much improved, how would you rate the change in your overall experience of running a neurofeedback session after implementing Lean methodology?"

Question 3: “Why did you select this rating?”

## Results

### Total time of a neurofeedback session

Before the implementation of Lean methodology, researchers could complete a neurofeedback session in 142 and 147 minutes respectively. After the implementation of Lean, they were able to complete a neurofeedback session in 124 and 128 minutes respectively. This demonstrates that after Lean implementation, the EEG neurofeedback sessions were approximately 13% faster. This was largely because of reduced time spent during lab set-up and participant EEG cap preparation (total time pre-Lean for the 2 participants: 58 and 67 minutes, total time post-Lean: 46 and 38 minutes).

### Team member satisfaction

Six out of seven members of the EEG neurofeedback team filled out the anonymous internet survey. When asked how they would rate the changes in reducing time spent on set-up, running, and clean-up of a neurofeedback session, five out of six of the respondents selected “much improved” while 1 team member reported that they would rate it as “somewhat improved.” All team members considered their overall experience of running a neurofeedback session as “much improved”. Five out of 6 team members provided an explanation as to why they provided this answer. Responses included examples like the “The sessions run smoother, faster, and the protocol is very clear.” (Respondent 1); “When there are issues with software or setup, there is much more reference for trouble-shooting and answers readily available on the protocol. This allows for quicker set-up when there are issues that arise” (Respondent 2); “Everything runs so much smoother and faster.” (Respondent 4); “After the implementation of Lean, I have found that neurofeedback sessions are shorter.” (Respondent 5). These findings show that the runs were conducted faster after implementation of Lean methodology. The complete results from the anonymous internet survey are shown in Table 1.

**Table 1:** Team member responses to anonymous internet questionnaire.

**Q1 –On a scale of much worsened, somewhat worsened, the same, somewhat improved, much improved, how would you rate the changes in achieving the following goals by implementing Lean methodology (n = 6)?**					
	**Much worsened**	**Somewhat worsened**	**The same**	**Somewhat improved**	**Much improved**
Reducing time spent on set-up, running, & clean-up of a neurofeedback session	0	0	0	1 (17%)	5 (83%)
Streamlining data capture & storage	0	0	0	2 (33%)	4 (67%)
Setting the team up for continuous improvement initiatives	0	0	0	1 (17%)	5 (83%)

**Q2 –On a scale of much worsened, somewhat worsened, the same, somewhat improved, much improved, how would you rate the change in your overall experience of running a neurofeedback session after implementing Lean methodology (n = 6)?**				
**Much worsened**	**Somewhat worsened**	**The same**	**Somewhat improved**	**Much improved**
0	0	0	0	6 (100%)

**Q3 –Why did you select this rating for the previous questions (n = 5)?**	
Respondent 1	The sessions run smoother, faster, and the protocol is very clear.
Respondent 2	Improved paper protocol The overall layout of the protocol is very easy to follow, with step-by-step protocol on the left and corresponding details and troubleshooting on the right. Scripts were cut from the protocol packet, saving significant amounts of paper and making it easier to find steps (due to packet being half the size of the original). The overall time seems about the same for set-up when everything goes right. However, when there are issues with software or set-up, there is much more reference for troubleshooting and answers readily available on the protocol. This allows for quicker set-up when there are issues that arise. More visual references of icons and direct, copied images make it easier to follow steps (I think particularly beneficial for individuals who are new to the study and the protocol). ‘Co-running’ the runs on Upekkha* (lead scientist = mouse, EEG tech = keyboard) More fluid process, and I think potentially encourages higher focus and engagement from each team member due to the interactive process (each member’s next step relies on the other to complete first). I have personally found myself more aware of my own role while completing the runs, and also more aware of the other researcher’s role (thereby also potentially decreasing the frequency of mistakes from each individual). Debrief I think the debrief has been a great way to create an efficient medium to communicate issues, success, areas for improvement or areas that require attention, etc. Also encourages in-the-moment assessment of issues and protocol and encourages researchers to be aware of what happens during the runs, what is successful and what can be improved upon.
Respondent 3	[I chose it] because the workflow in the lab has become smoother and more professional. In my opinion, the main contribution of the Lean methodology was to help us see the participant as [a] client and the scientific experiment as a community service. This work paradigm changes everything: the improvement is necessary not only to collect good data, but also to have participants satisfied and happy with the experience of meditating in the lab. For me, that is extremely valuable.
Respondent 4	Everything runs so much smoother and faster.
Respondent 5	After the implementation of Lean, I have found that NF sessions are shorter and the research team has an increased understanding of the process. The debriefing process allows us to check in after the session and as a result, continuously improve based on feedback from all team members.

*Upekkha: name of computer used for data collection

EEG: electroencephalography

NF: neurofeedback

## Discussion

To our knowledge, this is the first study to apply Lean methodology to neuroimaging research. As expected, time to run the experiment was reduced and team member satisfaction was increased after the implementation of Lean.

Overall, these results show the potential usefulness of applying Lean thinking to neuroimaging research. Importantly, debriefing after each experiment to identify new ways to increase efficiency continued to prove effective even after the initial Lean process was completed. We have rolled out this continuous improvement approach to all other studies in our lab and have found that this focus has greatly changed the mindset of lab members. Everyone is now aware that even small improvements in efficiency add up and that everyone’s input and ideas are valued.

In the present study, we focused the application of Lean thinking to neurofeedback sessions as this part of the study was identified as containing the most ‘waste.’ A value stream map was chosen over other available tools because it allowed the team to visualize and discuss the entire workflow. This was deemed more useful as an introductory exercise than other tools (such as root-cause analysis and FMEA) because it helped the team self-identify specific area(s) on which to focus. It is also evident that Lean thinking can be applied to other areas of the research endeavor, including participant recruitment, data backup processes, and even the grant application process, and the tools used may vary. We speculate that adopting Lean thinking might even increase reproducibility and replicability of findings across studies through consistent workflows and a decrease in expenditure of energy and resources on processes that are not essential. Non-essential processes could add noise to the data acquisition process (e.g., by increasing stress levels in research technicians responsible for data acquisition which can lead to mistakes).

### Recommendations

To improve the processes and efficiency across academic research, it may be worthwhile to provide training on Lean methodology within post-doctorate programs. This will allow investigators to think critically about their internal operations and optimize their use of time, space, and funding to continuously improve their efficiency. A “return on investment” will likely be obtained as increases in efficiency, team member satisfaction and team cohesion will be far greater than the time taken to apply the methodology. In addition to providing training, it may also be worthwhile to consider enlisting an external consultant to work directly with the research team.

### Limitations

A major limitation of the present study is the low sample size. However, the finding of reduced time of running a neurofeedback session for the two participants before versus after Lean implementation was corroborated by the anonymous internet survey results, with 5 out of 6 EEG neurofeedback team members answering that the reduction of time spent on the neurofeedback sessions was much improved (and 1 team member stating it was somewhat improved). In addition, 4 out of the 5 team members who provided additional feedback used wording indicating that the runs were faster (“The sessions run smoother, faster, and the protocol is very clear.” (Respondent 1); “when there are issues with software or setup, there is much more reference for trouble-shooting and answers readily available on the protocol. This allows for quicker set-up when there are issues that arise” (Respondent 2); “Everything runs so much smoother and faster.” (Respondent 4); “After the implementation of Lean, I have found that neurofeedback sessions are shorter.” (Respondent 5). These findings provide confidence in the current findings of a reduction in time after Lean implementation. Still, the findings should be considered preliminary and warrant replication.

Another limitation of the current study is that Lean methodology was applied to an ongoing research study, which meant that active components of the protocol could not be changed. Ideally this type of process improvement would be implemented before the start of a study which would allow for changes to be made to the research protocol without impacting data collection.

In sum, this is the first application of Lean methodology to neuroimaging. The results imply that the implementation of Lean tools such as value stream mapping, root-cause analysis, and 5S can eliminate inefficiencies and help set a research team up for continuous improvement.

## Supporting information

S1 File Materials and methods section for randomized clinical neuroimaging trial. (DOCX)
