# Supplementary material for: Improving efficiency in neuroimaging research through application of Lean principles
Source: PLoS One. 2018 Nov 28;13(11):e0205232. doi: 10.1371/journal.pone.0205232 (PMC6261412; doi:10.1371/journal.pone.0205232)
Supplement: S1 File — (DOCX) [file pone.0205232.s001.docx]

**S1 File. Materials and methods section for randomized clinical neuroimaging trial**

**Materials and methods**

**Participants**

Sixty-three participants (21 males, 42 females, average age 47 + 10.88) were recruited from orientation sessions of the Mindfulness Based Stress Reduction ([MBSR](https://www.sciencedirect.com/topics/medicine-and-dentistry/mindfulness-based-stress-reduction)) program at the University of Massachusetts Medical School (UMMS) Center for [Mindfulness](https://www.sciencedirect.com/topics/neuroscience/mindfulness). Inclusion criteria were: (i) no current or past neurological disorders; (ii) ability to understand the study procedures and a willingness to commit to the demands of the study protocol; (iii) stable dose of psychotropic medications for at least three months; (iv) fluency in English; (v) willingness to remain in the area for the duration of the study; (vi) taking the MBSR course. Exclusion criteria were: (i) prior participation in an MBSR course; (ii) regular meditation practice which was defined as meditating for more than 30 minutes per day over five days in the two months prior to beginning assessments or attending a retreat that was five-days or longer in the two years prior to beginning assessments; (iii) current or past serious psychiatric, cognitive, or medical disorder; (iv) unstable dose of psychotropic medication or use of anti-psychotic medication or stimulants; (v) current alcohol use that exceeded fourteen drinks per week or four drinks at any one time for males and more than seven drinks per week or three drinks at any one time for females; (vi) substance abuse or dependence six months prior to baseline or illegal drug use six weeks prior to beginning assessments; (vii) claustrophobia; (viii) structural brain damage; (ix) functional magnetic resonance imaging (fMRI) incompatible implants; (x) adults unable to consent; (xi) minors; (xii) pregnant women; (xiii) prisoners. Demographics are shown in Table S1. The study was approved by the UMMS Institutional Review Board.

| **S1 Table. Demographics.** | | | | |  |
| --- | --- | --- | --- | --- | --- |
|  | Control  (*N* = 30) | Active  (*N* = 33) | Test statistics | *P* | |
| Gender (male/female) | 10/20 | 11/22 | 0 | 1 | |
| Age (mean with standard deviation in parentheses) | 45 (12) | 48 (10) | 413 | 0.262 | |
| Highest level of completed education (college or university/graduate school) | 11/16 | 9/20 | 441.5 | 0.440 | |
| Work status (full-time/part-time/not in labor force/unemployed) | 17/7/4/2 | 21/4/4/4 | 2.900 | 0.638 | |
| Marital status (never married/married/living in permanent relationship/separated/divorced) | 8/10/4/5/3 | 4/19/4/1/5 | 6.970 | 0.133 | |
| Race (White/African American/Asian/Hispanic/White and Hispanic/White and African American) | 27/0/2/0/0/0/1 | 28/0/3/1/1/1/0 | 2.957 | 1 | |

Differences in gender were tested using the chi-square test. As the assumptions of the chi-square test did not hold for work status, marital status and race, these variables were tested using Fisher’s exact tests. Highest completed level of education was tested using the Mann-Whitney test. Differences in age was also tested using the Mann-Whitney test because of a non-normal distribution.

**Design**

All participants followed an 8-week Mindfulness Based Stress Reduction Course and were randomized to receive five sessions of meditation with electroencephalography (EEG) neurofeedback from the posterior cingulate cortex (PCC; Active group) or meditation with EEG but without neurofeedback (Control group) at week 3, 4, 5, 6 and 7. At baseline in the two weeks before the first class, brain activity in the PCC during meditation was assessed in an fMRI experiment, attention was measured using a Rapid Visual Information Processing (RVIP) task, perceived stress was measured using the Perceived Stress Scale (PSS), and physical, mental and social health was recorded with a Patient Reported Outcomes Measurement Information System (PROMIS) questionnaire. These measures were repeated within two weeks after the last MBSR class. At three months after the last class, participants completed the PSS and PROMIS questionnaire to investigate long-term effects. Fig S1 shows a schematic representation of the design of the study and extended information about each measure as well as the intervention is provided below.

**S1 Fig. Study design of randomized clinical neuroimaging trial**

**
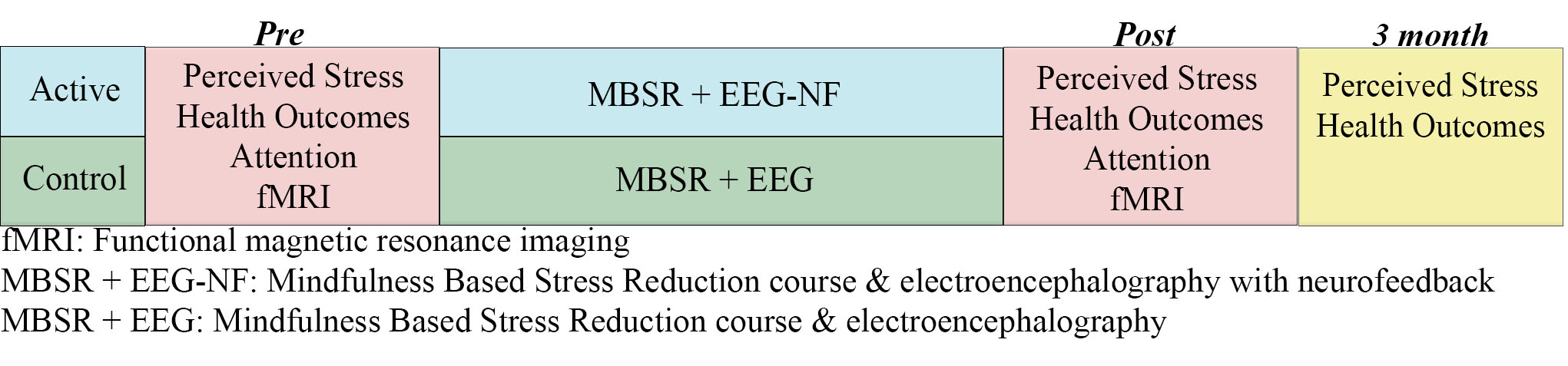
**

**Intervention**

**Mindfulness Based Stress Reduction**

The MBSR program consisted of eight weekly 2.5-hour sessions and an all-day session in week 6. It included training in formal [meditation](https://www.sciencedirect.com/topics/medicine-and-dentistry/meditation) practices like body scan, sitting meditation, walking meditation, and mindful movement, as well as informal practices to integrate mindfulness into everyday life [1]. Participants were encouraged to practice for 45 min at home 6 days per week. The amount of time spent on home practice per day was recorded.

**EEG**

Both the active and the control group electroencephalography data were recorded with a high-density EEG system using a cap with 128 active electrodes (BioSemi, Amsterdam, the Netherlands) and signals were digitized on-line by a computer at a rate of 2048 Hz.

Both the active and the control groups performed five EEG sessions (at week 3, 4, 5, 6 and 7 of the MBSR course). Before the first session at week 3, each participant was instructed on breath awareness meditation and how to use the neurofeedback signal to guide their meditation practice. During each session, participants performed six runs of breath awareness meditation with their eyes closed. Each run started with 20 seconds of resting state, followed by 3 minutes and 40 seconds of meditation. For all sessions, participants were asked after run 2 and run 4 to describe what they did during the two previous runs during the resting state and the meditation part of the runs to verify accurate performance. Each session started with a practice session to help participants get used to meditating in a research setting.

*Active group*

Real-time neurofeedback was provided using an auditory version of a visual neurofeedback paradigm that provides neurofeedback from the posterior cingulate cortex (PCC) [2]. Briefly, the real-time neurofeedback module applied an average reference to the incoming EEG signal, after which the EEG was band-pass filtered between 40 and 57 Hz using a 2nd order infinite impulse response (IIR) Butterworth filter. This frequency band was chosen based on electro and magnetoencephalographic findings of task-related gamma-band power suppression in the posterior cingulate cortex (PCC) and pilot testing [3–5]. PCC activity (MNI coordinates−6,−60, 18) was estimated using a spatial filter constructed in accordance with the linearly constrained minimum variance (LCMV) beamformer technique by means of EMSE suite (Source Signal Imaging, La Mesa, CA, USA) and neurofeedback was provided using an in-house developed software [6,7]. Beamformers have been successfully used in previous EEG studies assessing brain activity in the PCC [8,9]. PCC neurofeedback was provided using 32 active electrodes (BioSemi, Amsterdam, the Netherlands) whose scalp locations were identified using a sensor-based dimensionality reduction method for high-density neurofeedback systems. Signals were digitized on-line by a computer at a rate of 2048 Hz. The PCC coordinates were defined based on peak deactivation in our previous study of meditation and real-time fMRI neurofeedback from these coordinates has been shown to correlate with the subjective experience of effortless awareness [10,11]. A realistic (average) head model with different electrical conductivities for skull, scalp and brain was employed [12]. This approach has been shown to improve source estimation compared to similar spherical head models [13]. Each second, PCC signal power was calculated as the root mean square of the 40–57 Hz band filtered PCC activity by averaging within 1 s segments. During the effortless awareness part of each task, the PCC signal power in each segment was baseline corrected by subtracting mean PCC activity during baseline and dividing by the standard deviation during baseline. After this, segments were smoothed by applying a half-Gaussian curve, multiplying the last data point by 0.57, the preceding data point by 0.35 and the second to last data point by 0.08. The feedback signal was presented auditory over loudspeakers and updated every 2 seconds.

To help participants learn how to use the neurofeedback signal to guide their meditation, a graphical representation of the auditory feedback was presented after each run. Participants were asked “Is there anything noteworthy that you notice about how this graph or the auditory feedback relate to your experience of effortless awareness during the meditation?” to help them reflect on the nature of the link between their PCC activity and the quality of their meditation.

*Control group*

At the beginning of each session, participants received a reminder on how to perform breath awareness meditation. Each session started with a practice session to help participants get used to meditating in a research setting. Participants received the same number of breath awareness meditation runs as the active group while their EEG was measured. However, they did not receive any neurofeedback.

**Measures**

**Behavioral**

*Perceived Stress Scale (PSS)*

Stress appraisal was measured using the 10-item version of Perceived Stress Scale (PSS-10) [14]. Specifically, the PSS-10 measures the degree to which one perceives aspects of one's life as stressful. This measure has a 5-item Likert scale with possible responses ranging from 0 (never) to 4 (very often), indicating how often they have felt within the past month. Positively worded items are reversed scored. Total scores range from 0 to 40, with higher scores indicating greater perceived stress. The PSS-10 has good internal consistency (Cronbach’s alpha estimates ranging between 0.74 and 0.91) and test-retest reliability (*r* = 0.77 and ICC = 0.86) and PSS scores are sensitive to change following MBSR [15]. In addition, studies have found significant associations between perceived stress as assessed by the PSS-10 and anatomical brain changes following mindfulness training [16].

**Exploratory**

*Patient Reported Outcomes Measurement Information System (PROMIS)*

Physical, mental and social health was recorded with the Patient Reported Outcomes Measurement Information System (PROMIS) 29 item questionnaire version 1. It measures seven domains including fatigue, depression, anxiety, sleep disturbance, physical function, satisfaction with social role, and pain interference as well as one 11-point rating scale for pain intensity [17]. Norm-based scores have been calculated for each domain, such that a score of 50 represents the mean of the general population (standard deviation=10). High scores represent more of the domain being measured. Thus, on symptom-oriented domains of PROMIS-29 (anxiety, depression, fatigue, pain interference, and sleep disturbance), higher scores represent worse symptomatology. On the function-oriented domains (physical functioning and social role) higher scores represent better functioning. Face and construct validity of this measure is given by the process for development of an extensive item bank, which was based on comprehensive literature searches of existing instruments, qualitative item analysis, consensus building, and recent and ongoing studies that have provided data to establish the equivalency of PROMIS 29 scales with “legacy” measures [18].

*RVIP*

Sustained attention and working memory was measured using a Rapid Visual Information Processing (RVIP) task. Response times and detection accuracy from this task have been previously correlated with DMN function [19]. The RVIP is administered using custom software, implemented in PsychoPy, which conforms to the literature describing its original implementation in the Cambridge Neuropsychological Test Automated Battery [20,21]. A pseudorandom stream of digits (0-9) is presented to the participants in white, centered on a black background, surrounded by a white box. Participants are instructed to press the space-bar whenever they observe the sequences 2-4-6, 3-5-7, or 4-6-8. Digits are presented one after another at a rate of 100 digits per minute and the number of stimuli that occurred between targets varied between 8 and 30. Responses that occurred within 1.8 seconds of the last digit of a target sequence being presented were considered “hits". Stimuli presentation continued until a total of 32 target sequences were encountered, which required on average 4 minutes and 20 seconds. Before performing the task, participants completed a practice version that indicated when a target sequence occurred.

Responses that occurred within 1.8 seconds of the last digit of a target sequence being displayed were considered hits, multiple responses within 1.8 seconds were considered a hit followed by multiple false alarms, and responses that occur outside of the 1.8-second window were considered false alarms. The number of hits and false alarms were converted to rates by dividing by the total number of targets. Since the number of false alarms were not bounded, the false alarm rate can be higher than 100%, resulting in *A’* values greater than 1. In post-hoc analysis, false alarm raters greater than 1 were replaced with 1 and *A’* values greater than 1 were replaced with 0.

Summary statistics calculated from the RVIP included: mean reaction time, total targets, hits, misses, false alarms, hit rate *(H)*, false alarm rate *(F)*, and *A*. *A’* is an alternative to the more common *d’* in signal detection theory.

**fMRI**

*Acquisition*

Imaging was performed on a Philips Achieva 3 Tesla MRI scanner (Philips Medical Systems, Best, the Netherlands) at the UMMS Advanced MRI Center. Before the functional scans, a high-resolution anatomical scan was acquired (181 (sagittal) slices, repetition time(TR)/echo time (TE): 7.0/3.2 ms, shot interval: 3000 ms, field of view (FOV) 240 x 240 x 181, matrix = 240x240; 1 mm isotropic voxels) to improve localization of the functional data. After this, three body scan runs and three breath awareness runs were acquired with the following parameters: (139 scans, 37 (transverse) slices; TR/TE: 2000/30 ms, FOV 216 x 216 x 130, matrix = 80x80; 2.7x2.7x3.5 mm voxels). During these runs, participants were instructed to keep their eyes closed. Each run consisted of three parts: 60 seconds of resting-state, 38 seconds of instructions on the upcoming body scan or breath awareness meditation and 180 seconds of meditation.

**References**

1. Nehra DK, Nehra S, Dogra R. Biopsychosocial Issues in Positive Halth Positive Psychological Functioning with Mindfulness Based Stress Reduction ( MBSR ) Program. 2006;

2. van Lutterveld R, Houlihan SD, Pal P, Sacchet MD, McFarlane-Blake C, Patel PR, et al. Source-space EEG neurofeedback links subjective experience with brain activity during effortless awareness meditation. Neuroimage. Elsevier; 2017;151: 117–127. doi:10.1016/j.neuroimage.2016.02.047

3. Brookes MJ, Woolrich M, Luckhoo H, Price D, Hale JR, Stephenson MC, et al. Investigating the electrophysiological basis of resting state networks using magnetoencephalography. Proc Natl Acad Sci. 2011;108: 16783–16788. doi:10.1073/pnas.1112685108

4. Jerbi K. Exploring the electrophysiological correlates of the default-mode network with intracerebral EEG. Front Syst Neurosci. 2010;4: 1–9. doi:10.3389/fnsys.2010.00027

5. Ossandon T, Jerbi K, Vidal JR, Bayle DJ, Henaff M-A, Jung J, et al. Transient Suppression of Broadband Gamma Power in the Default-Mode Network Is Correlated with Task Complexity and Subject Performance. J Neurosci. 2011;31: 14521–14530. doi:10.1523/JNEUROSCI.2483-11.2011

6. Greenblatt RE, Ossadtchi A, Pflieger ME. Local linear estimators for the bioelectromagnetic inverse problem. IEEE Trans Signal Process. 2005;53: 3403–3412. doi:10.1109/TSP.2005.853201

7. Sekihara K, Nagarajan S, Poeppel D, Miyashita Y. Reconstructing spatio-temporal activities of neural sources from magnetoencephalographic data using a vector beamformer. 2001 IEEE Int Conf Acoust Speech, Signal Process Proc (Cat No01CH37221). 2001;3: 2021–2024. doi:10.1109/ICASSP.2001.941346

8. Höfle M, Pomper U, Hauck M, Engel AK, Senkowski D. Spectral signatures of viewing a needle approaching one’s body when anticipating pain. Eur J Neurosci. 2013;38: 3089–3098. doi:10.1111/ejn.12304

9. Michels L, Muthuraman M, Lüchinger R, Martin E, Anwar AR, Raethjen J, et al. Developmental changes of functional and directed resting-state connectivities associated with neuronal oscillations in EEG. Neuroimage. 2013; doi:10.1016/j.neuroimage.2013.04.030

10. Brewer JA, Worhunsky PD, Gray JR, Tang Y-Y, Weber J, Kober H. Meditation experience is associated with differences in default mode network activity and connectivity. Proc Natl Acad Sci. 2011;108: 20254–20259. doi:10.1073/pnas.1112029108

11. Garrison KA, Santoyo JF, Davis JH, Thornhill TA, Kerr CE, Brewer JA. Effortless awareness: using real time neurofeedback to investigate correlates of posterior cingulate cortex activity in meditators’ self-report. Front Hum Neurosci. 2013;7: 1–9. doi:10.3389/fnhum.2013.00440

12. Salu Y, Cohen LG, Rose D, Sato S, Kufta C, Hallett M. An Improved Method for Localizing Electric Brain Dipoles. IEEE Trans Biomed Eng. 1990; doi:10.1109/10.55680

13. Cuffin NB. EEG localization accuracy improvements using realistically shaped head models. IEEE Trans Biomed Eng. 1996; doi:10.1109/10.486287

14. Cohen S, Williamson G. Perceived stress in a probability sample of the United States [Internet]. The Social Psychology of Health. 1988. pp. 31–67. doi:10.1111/j.1559-1816.1983.tb02325.x

15. Lee EH. Review of the psychometric evidence of the perceived stress scale. Asian Nurs Res (Korean Soc Nurs Sci). Elsevier; 2012;6: 121–127. doi:10.1016/j.anr.2012.08.004

16. Hölzel BK, Carmody J, Evans KC, Hoge EA, Dusek JA, Morgan L, et al. Stress reduction correlates with structural changes in the amygdala. Soc Cogn Affect Neurosci. 2010;5: 11–17. doi:10.1093/scan/nsp034

17. Ader DN. Developing the patient-reported outcomes measurement information system (PROMIS). Med Care . 2007;45: S1e2.

18. Pilkonis PA, Yu L, Dodds NE, Johnston KL, Maihoefer CC, Lawrence SM. Validation of the depression item bank from the Patient-Reported Outcomes Measurement Information System (PROMIS®) in a three-month observational study. J Psychiatr Res. 2014; doi:10.1016/j.jpsychires.2014.05.010

19. Pagnoni G. Dynamical Properties of BOLD Activity from the Ventral Posteromedial Cortex Associated with Meditation and Attentional Skills. J Neurosci. 2012;32: 5242–5249. doi:10.1523/JNEUROSCI.4135-11.2012

20. Peirce JW. PsychoPy-Psychophysics software in Python. J Neurosci Methods. 2007;162: 8–13. doi:10.1016/j.jneumeth.2006.11.017

21. Sahakian BJ, Owen a M. Computerized assessment in neuropsychiatry using CANTAB: discussion paper. R Soc Med. 1992;85: 399–402. doi:10.1177/014107689208500711
